# Icariside II overcomes TRAIL resistance of melanoma cells through ROS-mediated downregulation of STAT3/cFLIP signaling

**DOI:** 10.18632/oncotarget.10582

**Published:** 2016-07-13

**Authors:** Juan Du, Jinfeng Wu, Xiuqiong Fu, Anfernee Kai-Wing Tse, Ting Li, Tao Su, Zhi-Ling Yu

**Affiliations:** ^1^ Consun Chinese Medicines Research Centre for Renal Diseases, School of Chinese Medicine, Hong Kong Baptist University, Kowloon Tong, Hong Kong; ^2^ Department of Chinese Medicine, Changhai Hospital, The Second Military Medicine University, Shanghai, China; ^3^ Department of Dermatology, Huashan Hospital, Fudan University, Shanghai, China

**Keywords:** melanoma, TRAIL, Icariside II, pSTAT3, ROS

## Abstract

Tumor necrosis factor-related apoptosis-inducing ligand (TRAIL) is a promising antitumor agent. However, many melanoma cells show weak responses to TRAIL. Here, we investigated whether Icariside II (IS), an active component of *Herba Epimedii*, could potentiate antitumor effects of TRAIL in melanoma cells. Melanoma cells were treated with IS and/or TRAIL and cell death, apoptosis and signal transduction were analyzed. We showed that IS promoted TRAIL-induced cell death and apoptosis in A375 melanoma cells. Mechanistically, IS reduced the expression levels of cFLIP in a phospho-STAT3 (pSTAT3)-dependent manner. Ectopic expression of STAT3 abolished IS-induced cFLIP down-regulation and the associated potentiation of TRAIL-mediated cell death. Moreover, IS-induced reactive oxygen species (ROS) production preceded down-regulation of pSTAT3/cFLIP via activating AKT, and the consequent sensitization of cells to TRAIL. We also found that IS treatment down-regulated cFLIP via ROS-mediated NF-κB pathway. In addition, IS converted TRAIL-resistant melanoma MeWo and SK-MEL-28 cells into TRAIL-sensitive cells. Taken together, our results indicated that IS potentiated TRAIL-induced apoptosis through ROS-mediated down-regulation of STAT3/cFLIP signaling.

## INTRODUCTION

Malignant melanoma is a highly aggressive and treatment-resistant cancer, with increasing incidence and high mortality rates worldwide. The 5-year survival rate of advanced melanoma was less than 10% [1]. Primary melanoma without any evidence of metastases is mostly treated by surgery. Chemotherapeutic agents such as dacarbazine and temozolomide (alkylating agents), targeted drugs such as vemurafenib and dabrafenib (targeting the BRAF^V600E^ mutation) for melanoma are currently used in the clinic, CTLA-4 and PD-1 antibodies represent an effective treatment option for metastatic melanoma and other cancer entities [2, 3]; however, they are not suitable for many patients because of toxicity, lack of the BRAF^V600E^ mutation, and/or developmentof resistance. It has been reported that ipilimumab, pembrolizumab and nivolumab can induce immune-related adverse events involving skin, gastrointestinal tract, liver, the endocrine system and other organ systems [2, 3–4]. Therefore, novel treatment strategies are still required.

Tumor necrosis factor-related apoptosis-inducing ligand (TRAIL/Apo2L), a type II transmembrane protein from the tumor necrosis factor superfamily, has been shown to selectively kill cancer cells and spares normal cells [5–7]. However, some cancer cells are highly resistant to TRAIL-induced apoptosis. Many factors may influence the sensitivity of these cancer cells to TRAIL. For example, overexpression of the cellular FLICE-like inhibitory protein (cFLIP) can attenuate death receptor-mediated apoptosis [8] by interfering with the activation of caspase-8 at the death inducing signaling complex (DISC). Suppression of cFLIP is sufficient to sensitize human melanoma cells to TRAIL-mediated apoptosis [9]. In addition, other reports have indicated that the role of c-FLIP in cancer cells is anti-apoptotic and interference with cFLIP expression could sensitize tumor cells to death ligands and chemotherapy in experimental models [10, 11].

A number of reports have suggested that cFLIP could be up-regulated by activated STAT3 [12, 13]. STAT3 plays a critical role in transcriptional regulation of genes that are involved in cell proliferation and survival. STAT3 can be activated by non-receptor tyrosine kinases such as Janus kinases (JAKs) or Src family kinases (SFKs) [14–17]. Under tyrosine phosphorylation, STAT3 homodimerizes or heterodimerizes with STAT1, then translocates to the nucleus, and binds to consensus DNA sequences within promoters of its target genes. Interestingly, it was reported that inhibition of PI3 kinase (PI3K) activity, which in turn inhibited AKT activation, significantly increased the DNA-binding activity of STAT3 in human glioblastoma U87and D54 cells without increasing STAT3 phosphorylation [18]. In contrast, another report suggested that constitutively active PI3K/AKT signal inhibited tyrosine phosphorylation of STATs [19].

Icariin is one of the major components of *Herba Epimedii*. Icariside II (IS) is a metabolite of icariin by the host microbiome [20]. It has been reported that IS induced apoptosis in various human cancer cell lines of different origins by targeting STAT3, PI3K/AKT, MAPK/ERK, COX-2/PGE2 and β-Catenin [21]. Moreover, IS has been documented to induce ROS and consequently trigger cell death [22]. ROS can affect the stability of cFLIP protein, thus impact sensitivity of cancer cells to TRAIL [23]. IS has been documented to attenuate streptozotocin-induced cognitive deficits and diabetes in rats. No obvious toxicity was observed in these rats after IS treatment. We hypothesized that IS might be a promising agent to enhance TRAIL response [24, 25]. Our data show that IS inhibits cFLIP expression and consequently potentiates TRAIL-induced apoptosis through ROS-dependent STAT3 and NF-κB inhibitions.

## RESULTS

### IS sensitizes A375 melanoma cells to TRAIL-mediated apoptosis

**Figure 1:**
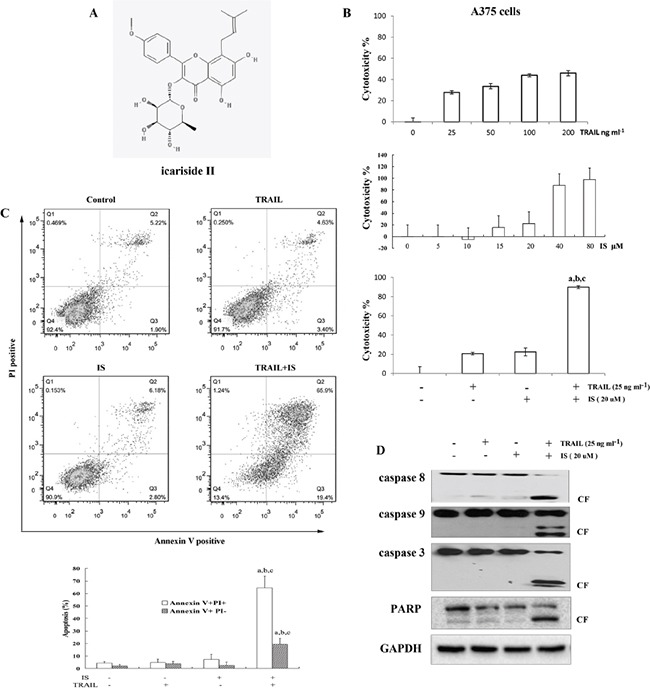
IS sensitizes TRAIL-induced apoptosis in A375 melanoma cells **A.** Chemical structure of IS. **B.** Cell viability was assessed by the MTT assay. **C.** Cells were stained with PI/Annexin V and then analyzed by FACS. **D.** Western blotting of lysates from A375 cells treated with IS and/or tumor necrosis factor–related apoptosis-inducing ligand (TRAIL) for 24 h using the indicated antibodies. ^a,b,c^*P*<0.05versus control(a), versus IS(b) and versus TRAIL(c). At least two independent experiments revealed largely comparable results.

We first examined the cytotoxic effects of IS and/or TRAIL. As expected, A375 melanoma cells displayed low response to TRAIL-induced cell death (Figure 1B, top). IS induced A375 cells death in a dose-dependent, while ≤ 20 μM IS showed low cytotoxiciy on A375 cells (Figure 1B middle). Interestingly, IS 20 μM significantly enhanced TRAIL-induced cytotoxicity, as determined by MTT assay (Figure 1B, bottom, Supplementary Figure S1A and Table 1). We also found that IS potentiated TRAIL-induced apoptosis assayed by annexin V/PI staining (Figure 1C). In addition, IS enhanced TRAIL-induced activation of caspase-3, −8, and −9, which in turn led to increased PARP cleavage (Figure 1D and Supplementary Figure S1B).

### IS down-regulated expression of cFLIP contributes to the enhancement of TRAIL-induced apoptosis

**Figure 2:**
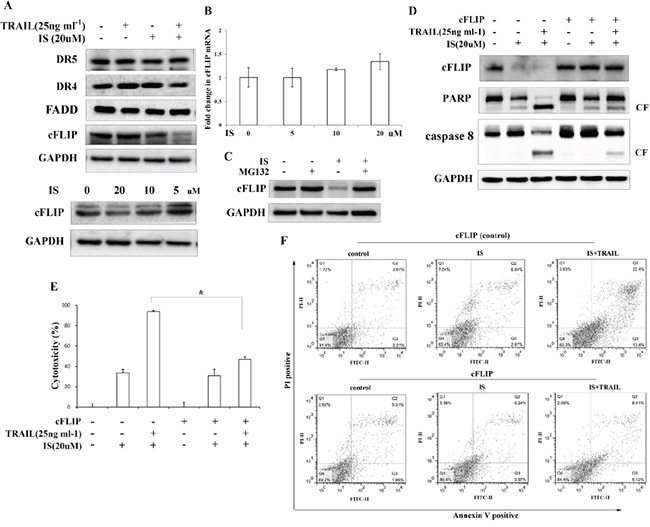
IS-induced cFLIP downregulation is essential for sensitization of TRAIL-mediated apoptosis **A.** Western blotting of DR5, DR4, FADD and cFLIP in A375 cells pretreated with IS and/or TRAIL for 24 h. **B.** Real-time PCR analysis of cFLIP mRNA. **C.** Western blotting of lysates from A375 cells pretreated with IS for 18 h followed by MG132 for 6 h using a cFLIP antibody. Cells were transiently transfected with a cFLIP plasmid, followed by IS and/or TRAIL treatment for 24 h. **D.** Cell extracts were prepared for Western blot analysis of cFLIP, caspase 8 and PARP. **E.** A375 cells viability was assessed by the MTT assay. *P<0.05. **F.** cFLIP-transfected cells were stained with PI/Annexin V and then analyzed by FACS. At least two independent experiments revealed largely comparable results.

To determine the mechanism of combined effects of IS and TRAIL, we next examined the expression levels of TRAIL receptors and TRAIL DISC proteins, including FADD and c-FLIP, in IS-treated cells. A375 cells were treated with IS and/or TRAIL for 24 h and the proteins were analyzed by Western blotting. We observed that IS did not increase the expressions of DR4, DR5 and FADD in A375 cells (Figure 2A, top). However, IS down-regulated cFLIP in a dose-dependent manner (Figure 2A, bottom). We next analyzed how cFLIP protein expression is inhibited by IS. Using realtime RT-PCR, we found that IS did not decrease cFLIP mRNA expression (Figure 2B), suggesting that IS does not reduce cFLIP expression at transcriptional level. Next we examined cFLIP protein expression in cells treated with MG132, a proteasome inhibitor. As shown in Figure 2C, cFLIP protein expression levels were restored in cells co-treated with IS and MG132, strongly suggesting that IS induces cFLIP downregulation through proteasome-mediated protein degradation.

We next examined whether exotic expression of cFLIP could abrogate the sensitization of IS on TRAIL-induced cytotoxicity and apoptosis. As shown in Figure 2D, IS could not decrease cFLIP expression in cFLIP-overexpressed cells. Caspase 8 and PARP cleavage induced by IS plus TRAIL were significantly decreased in cFLIP-overexpressed cells as compared to vector-transfected cells (Figure 2D). The cytotoxicity induced by IS plus TRAIL was also decreased in cFLIP-overexpressed cells (Figure 2E). Moreover, the effect of IS on TRAIL-induced cell apoptosis was markedly abolished in cells transfected with cFLIP (Figure 2F), as compared to that in vector-transfected cells.

### STAT3/AKT signaling is involved in IS-induced cFLIP down-regulation

**Figure 3:**
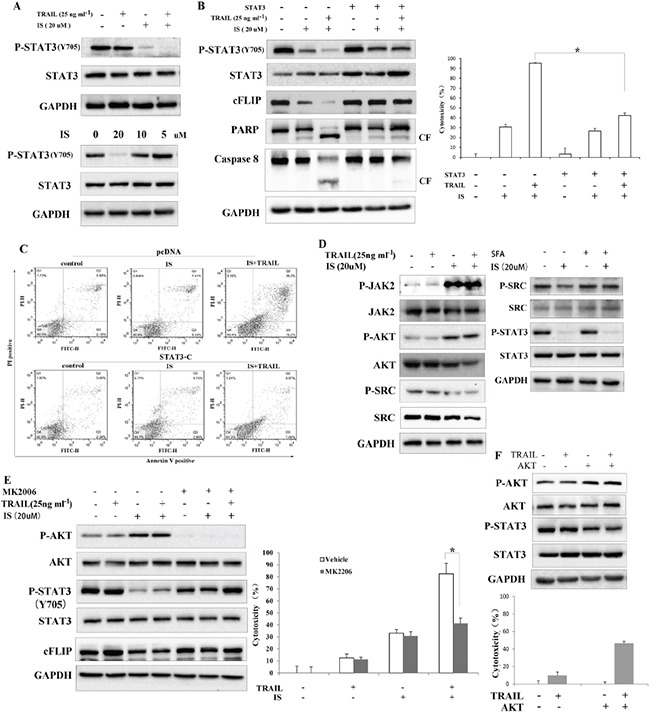
Inhibition of AKT-mediated STAT3 activation is required for IS-induced downregulation of cFLIP **A.** Western blotting of pSTAT3 and STAT3 in A375 cells. **B.** (left) Cells were transfected with constitutively activated STAT3 and Western blot analyses of pSTAT3, cFLIP, caspase 8 and PARP were performed. (Right) A375 cell viability was assessed by the MTT assay. *P<0.05. **C.** STAT3-transfected cells were stained with PI/Annexin V and then analyzed by FACS. **D.** (Left) Western blotting of STAT3 activation related components in A375 melanoma cells. (Right) ASrc activator, SFA doesnot affect the effects of IS on pSTAT3 expression. **E.** (Left) A JAK inhibitor, MK2206, abolishesthe downregulation of pSTAT3 and cFLIP in IS-treated cells; (Right) A375 cell viability was assessed by the MTT assay. *P<0.05. **F.** (Top) Cells were transfected with AKT and Western blot analyses of pSTAT3 and cFLIP were conducted; (Bottom) A375 cell viability was assessed by the MTT assay. *P<0.05. At least two independent experiments revealed largely comparable results.

Given the fact that cFLIP can be up-regulated by activated STAT3, we next investigated whether IS down-regulated cFLIP in a STAT3-dependent manner. As shown in Figure 3A, IS significantly inhibited the levels of phosphorylated STAT3 (Tyr 705) in a dose-dependent manner, and enhanced the inhibitory effects of TRAIL on pSTAT3 expressionas compared with TRAIL treatment alone, whereas protein levels of total STAT3 were not altered. We also observed that IS inhibited the expression of STAT3-targeted genes, such as survivin and Bcl-xl (Supplementary Figure S3). To further clarify whether the sensitizing effects of IS was mediated by STAT3, A375 cells were transiently transfected with an active form of STAT3 (STAT3-C) for 48 h, after that IS and/or TRAIL were added for another 24 h. As shown in Figure 3B, over-expression of STAT3-C abolished IS-induced cFLIP down-regulation and IS plus TRAIL-induced Caspase 8, PARP cleavage (Left) and cytotoxicity (right). In addition, the effect of IS plus TRAIL on apoptosis was partially abolished in cells transfected with STAT3-C (Figure 3C).

Next, to address how IS down-regulated pSTAT3 in melanoma cells, we investigated the role of non-receptor tyrosine kinases, protein phosphatases and protein kinases on IS-induced pSTAT3 down-regulation. Our data showed that IS increased the phosphorylation of JAK2 and AKT and decreased the phosphorylation of Src (Figure 3D, left), while Src activation could not reverse IS-induced decrease of pSTAT3 (right). Interestingly, an AKT inhibitor (MK2206, Figure 3E, left) reversed IS-induced down-regulation of pSTAT3 and cFLIP, suggesting that IS might down-regulate pSTAT3 via affecting protein kinase. In line with this, pretreatment with MK2206 abrogated the cytotoxicity induced by IS plus TRAIL (Figure 3E, right). Next, AKT was constitutively activated by AKT-overexpressed plasmid transfection in A375 cells. As shown in Figure 3F, activated AKT inhibited pSTAT3 (Top) and increased TRAIL-induced cell death (Bottom).

### IS potentiates TRAIL-induced apoptosis through ROS generation

**Figure 4:**
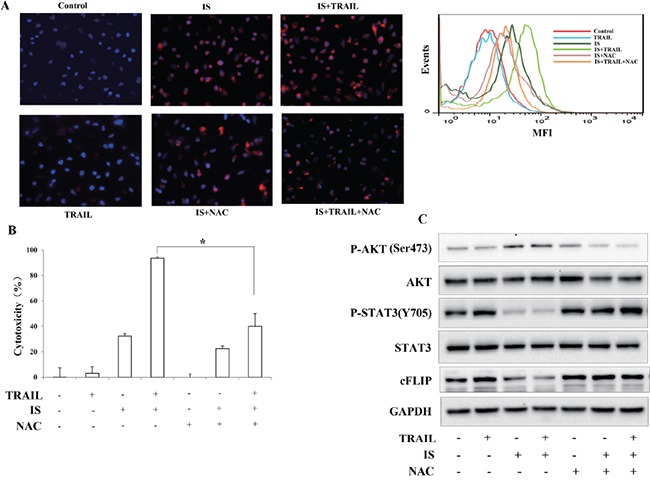
ROS mediates IS-induced downregulations of cFLIP and pSTAT3 **A.** (Left) Fluorescence was measured using fluorescent microscope with Cell ROX Deep Red Reagents for ROS, and DAPI for nuclei. (Right) Flow cytometry analysis of ROS. **B.** A375 cell viability was assessed by the MTT assay. *P<0.05. **C.** A375 cells were treated with IS and/or TRAIL in the presence/absence of 2 mM N-acetyl-L-cysteine (NAC). Then, Western blotting analysis was performed using anti-cFLIP, anti-pSTAT3 and anti-pAKT antibodies. At least two independent experiments revealed largely comparable results.

Many studies have shown that ROS plays a critical role in sensitizing melanoma to TRAIL treatment [26–28]. Thus, the production of ROS after IS and/or TRAIL treatments was examined. Using a specific fluorescent probe for ROS detection, a significant increase of ROS was observed in A375 cells after IS or IS plus TRAIL treatment for 6 h. In contrast, pretreatment with N-acetyl-L-cysteine (NAC), a ROS scavenger, effectively blocked IS-induced ROS production (Figure 4A, left). Similar tendency were observed by flow cytometry assays (Figure 4A, Right), IS and IS plus TRAIL induced-ROS generation was increased, as compared with medium control or TRAIL treatment alone group. We also found that NAC abrogated IS plus TRAIL-induced apoptosis (Figure 4B). ROS was documented to play an important role in regulation of cFLLP [23]. Therefore, we determined whether ROS is involved in IS-induced down-regulations of pSTAT3 and cFLIP. As shown in Figure 4C, treatment with NAC markedly reduced IS-induced AKT activation and reversed IS-induced pSTAT3 and cFLIP down-regulations.

### NF-κB inactivation is also involved in IS-induced cFLIP downregulation

**Figure 5:**
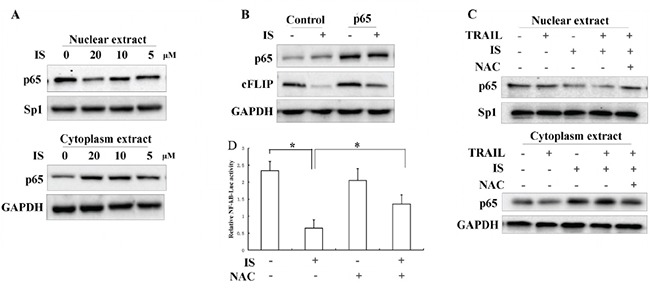
Inhibition of NF-κB contributes to the enhancement of TRAIL-induced apoptosis by IS **A.** A375 cells were treated with 20μM IS for 24 h; nuclear and total extracts were isolated as described in the Materials and Methods section and analyzed for p65, Sp1, and GAPDH by Western blotting. **B.** Western blotting analyses of transfected A375 cells using anti-p65 and anti-cFLIP antibodies. **C.** Western blotting analyses of A375 cells treated with IS and/or TRAIL in the presence or absence of NAC (2 mM). **D.** Luciferase activity of A375 cells transfected with pNF-kB-Luc and treated with 20 μM IS in the presence or absence of NAC (2 mM) for 24 hours. *P<0.05. At least two independent experiments revealed largely comparable results.

Previous studies showed that NF-κB inhibitor promoted TRAIL-induced apoptosis in a cFLIP-dependent manner [29, 30]. We observed that IS inhibited nuclear translocation of NF-κB p65 in a dose-dependent manner (Figure 5A and Supplementary Figure S5). Ectopic expression of p65 by transient transfection partially blocked IS-induced down-regulation of cFLIP (Figure 5B). Pretreatment with NAC significantly attenuated the inhibitory effect of IS on NF-κB p65 (Figure 5C). We also found that IS treatment markedly reduced the NF-kB-dependent luciferase activity, while pretreatment with NAC partially reversed this inhibitory effect (Figure 5D).

### IS sensitizes TRAIL-resistant melanoma MeWo and SK-MEL-28 cells to TRAIL

**Figure 6:**
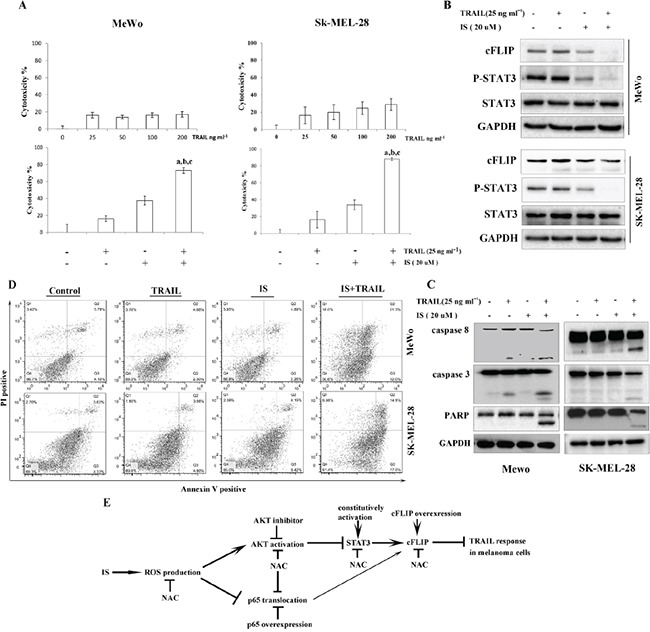
IS sensitizes TRAIL-resistant melanoma cells to TRAIL treatment **A.** Cell viability was assessed by the MTT assay. ^a,b,c^*P*<0.05 with a versus control, b versus IS and c versus TRAIL. **B.** Whole-cell extracts were analyzed for expression levels of cFLIP and pSTAT3 by Western blotting. **C.** Cells were stained with PI/Annexin V and then analyzed by FACS. **D.** Western blotting of lysates using the indicated antibodies. **E.** Schematic diagram of the mechanism by which IS potentiates TRAIL-induced apoptosis. At least two independent experiments revealed largely comparable results.

It has been reported that some melanoma cells such as MeWo and SK-MEL-28 cells are completely resistant to TRAIL [26–28, 31]. As expected, both MeWo and SK-MEL-28 cells had no responses to TRAIL(Figure 6A top). Treatment with IS significantly enhanced TRAIL-induced cytotoxicity, as determined by the MTT assay (Figure 6A bottom). Interestingly, the primary human skin fibroblast had no response to IS and/or TRAIL (Supplementary Figure S6). We also found that IS induced the downregulation of pSTAT3 and cFLIP in MeWo and SK-MEL-28 cells (Figure 6B). Further, we observed that treatment of MeWo and SK-MEL-28 cells with IS in combination with TRAIL resulted in markedly increased PARP, caspase-8 and caspase-3 cleavages (Figure 6C), and apoptosis (Figure 6D).

## DISCUSSION

cFLIP represents an attractive therapeutic target for melanoma, especially in combination with TRAIL receptor agonists. In this study, we demonstrated that IS significantly sensitized human melanoma cells to TRAIL-induced apoptosis through downregulation of cFLIP in a STAT3-dependent way. ROS production contributes to IS-induced decreasement of pSTAT3 via AKT activation. We also found that IS down-regulated cFLIP partially through inhibition of NF-κB nuclear translocation (summarized in Figure 6E). The anti-inflammatory, anticancer activities and the pharmacokinetic of IS have been studied [21, 32, 33]. IS might be a promising agent in reversing TRAIL resistance.

Our results indicated that IS sensitized TRAIL-induced apoptosis through the ROS-STAT3-cFLIP pathway. However, overexpression of cFLIP could not completely reverse TRAIL/IS-induced cell death (Figure 2E). The cytotoxicity probably was induced by IS alone as overexpression of cFLIP had no effect on IS-induced cell death (Figure 2D). It has been reported that IS induced apoptosis in various human cancer cell lines of different origins [21]. Notably, numerous reports have suggested that JAK2 and Src would act as activators for STAT3 [14–17]. In addition, SHP-1 and SHP-2 have also been reported to play crucial roles in inhibition of STAT3 in HCC [34–36]. However, our study indicated that AKT, but not others, is the major target of IS in sensitizing melanoma cells to TRAIL treatment. It has been reported that constitutive activation of PI3K/AKT signaling inhibits tyrosine phosphorylation of STATs [29, 30]. Ghosh MK et al. also reported that the PI3K/AKT pathway negatively regulated EGFR-dependent DNA-bindingactivity of STAT3 in glioblastoma multiforme cells [18]. No significant change in SHP-1 or SHP-2 expression was observed after IS treatment (Supplementary Figure S2). Although the activation of Src was inhibited by IS, Src activator could not reverse IS-induced inhibition of pSTAT3, indicating that Src might not be the upstream modulator of IS-mediated STAT3 inhibition. Interestingly, JAK2 was activated by IS. Since JAK2 plays an important role in mediating STAT3 activation [37, 38], IS combined with a JAK inhibitor might enhance TRAIL-induced apoptosis. Further investigations are necessary to be carried out to evaluate the combined effects of IS, JAK inhibitor and TRAIL.

We found that ROS plays a critical role in IS-induced down-regulation of cFLIP. Wilkie-Grantham et al. reported that ROS-dependent phosphorylation and ubiquitination of the c-FLIP protein causesd its proteasome-mediated degradation, thus sensitizing melanoma to TRAIL-induced cell death [23]. In this study, IS-induced cFLIP protein degradation was in a STAT3-dependent manner during oxidative stress. Exotic expression of STAT3 effectively reversed IS-induced down-regulation of cFLIP. Interestingly, ROS production in IS treatment alone group was markedly lower than that in IS plus TRAIL group. This can be explained by the following: (1) there is a relationship of mutual promotion between IS and TRAIL; and (2) IS induced autophagy and ROS at the same time. Autophagy may inhibit ROS accumulation [39, 40]. And TRAIL might inhibit IS-induced autophagy. Our data indicated that IS could induce LC3, a marker of autophagy, activation, while TRAIL could inhibit IS-induced LC3 activation. An autophagy inhibitor, 3-MA could increase IS-induced ROS production (Supplementary Figure S4), which suggested that autophagy might be involved in IS-induced ROS production. It has been reported that cellular and viral FLIPs suppress autophagy by preventing Atg3 from binding and processing LC3 [41]. In this respect, LC3 activation maybe attributed to IS induced down-regulaiton of cFLIP. Further investigation is needed to clarify the role of cFLIP in IS-induced autophagy.

Many studies have shown that cFLIP can be up-regulated by NF-κB activation and inhibition of nuclear translocation of NF-κB could effectively promote TRAIL-induced apoptosis via inhibiting cFLIP [42, 43]. In our system, we found that IS could inhibit p65 nuclear translocation in A375 melanoma cells and introduction of ectopic p65 partly restored the protein level of cFLIP in IS-treated cells. Furthermore, it has been shown that inhibition of nuclear translocation of NF-κB can be abolished by the antioxidant NAC(Figure 5C). Collectively, NF-κB pathway might be involved in IS-induced sensitization of melanoma cells to TRAIL treatment.

In conclusion, IS can enhance TRAIL sensitivity in melanoma cells via down-regulation of cFLIP, a well-known anti-apoptotic protein. These effects were partially mediated through ROS-STAT3 or NF-κB signaling. Combination of TRAIL and IS may be a potent therapy for melanoma, which warrants further *in vivo* studies.

## MATERIALS AND METHODS

### Reagents and antibodies

Icariside II (Figure 1A, IS, purity 99%)was purchased from Shanghai Ronghe Co. (Shanghai, China). IS at various concentrationswas dissolved in DMSO and then added to thecells in 5% fetal bovine serum (FBS)–containing DMEM. Recombinant TRAIL was purchased from PEPROTECH (Rocky Hill, NJ08553 USA). An AKT inhibitor, MK2206 was purchased from Selleck Chemicals LLC and a Src Family Activator (SFA) was purchased from Santa Cruz, CA. Antibodies against PARP, caspase-9, caspase-8, caspase-3, DR5, DR4, cFLIP, FADD (Fas-associated protein withdeath domain), c-FLIP (cellular FLICE-inhibitory protein), phospho-STAT3 (pSTAT3; Tyr705), STAT3, phospho-Akt (pAkt; Ser473), AKT, NF-κBp65 and LC3 A/B were purchased from Cell Signaling, MA. Antibody against DR4 was purchased from Santa Cruz, CA. MG132 and antioxidant N-acetyl-L-cysteine (NAC) werepurchased from Sigma-Aldrich (St. Louis, MO).

### Cell viability analysis

The effect of individual agents on cell viability wasassessed by using the MTT (3-(4,5-dimethylthiazol-2yl)-2,5-diphenyltetrazolium bromide; Life Technologies, Carlsbad, CA, USA) assay in six replicates.

### Annexin V/PI assay

The indicator of cell death and apoptosis was detected by usingannexin V/PI binding kit (Abcam, Cambridge, MA). Briefly, melanomacellswere treated with 20 μM IS and 25 ngml^−1^ TRAIL for 24 h. Then, cells were trypsinized, stainedwithannexin V/PI, and then analyzed with a flow cytometer.

### Western blot analysis

Whole-cell protein and nuclear lysates were prepared and analyzedby Western blotting as described previously [44].

### Transient transfection

Homo sapiens AKT1 gene was cloned by an RT-PCR product, which was amplified from total RNA extracted from SW480 human colon cancer cells. PCR primers were designed based on a published nucleotide sequence of human AKT1 (GenBank: accession no. AB451242.1). The primers are 5′ –TAGGATCCAGCGACGTGGCTATTGTGAAG–3′ (forward) and 5′ –TGAATTCTCAGGCCGTGCCGCTGG CCGAG–3′ (reverse). The gene was then cloned into mammalian expression vector pcDNA3.1 (Life Technology, NY, USA) at BamHI and EcoRIsites. The clone was sequenced to verify the authenticity of the gene. The pGL3-FLIP, constitutive activated STAT3 expression constructs (Stat3-C), GFP-RelAplasmids and the vector pcDNA used in this studywere obtained from Addgene(Cambrige, MA). Transfection of plasmids into melanoma cells was conducted by using Lipofectamine 2000 transfectionreagent (Invitrogen, Carlsbad, CA) following company's instruction. Cells were transfected with plasmids for 48 h before functional assays were carried out.

### Measurement of reactive oxygen species

Cells were plated on glass slides in 6-well plates. The cells were treated with 20 μM IS and/or 25 ngml^−1^ TRAIL in the presence/absence 2 mM NAC for 24 h at 37°C. The cells were then stained with 5 μM CellROX™ Green Reagent (Invitrogen, Carlsbad, CA, USA) and incubating at 37°C for 30 min. The cells were washed with PBS and imaged on a Leica DMI 3000 B inverted microscope using a 40x objective or analyzed by Flow cytometry.

### Statistical analyses

All data are expressed as mean ± SD of three independent experiments. Statistical significance was determined using unpaired Student's *t*-test and a *P*-value of less than 0.05 was considered statistically significant.

## SUPPLEMENTARY FIGURES AND TABLES


