# Supplementary material for: Icariside II overcomes TRAIL resistance of melanoma cells through ROS-mediated downregulation of STAT3/cFLIP signaling
Source: Oncotarget. 2016 Jul 13;7(32):52218–29. doi: 10.18632/oncotarget.10582 (PMC5239546; doi:10.18632/oncotarget.10582)
Supplement: Supplementary file 1 [file oncotarget-07-52218-s001.pdf]

Icariside II overcomes TRAIL resistance of melanoma cells through ROS-mediated downregulation of STAT3/cFLIP signaling

SUPPLEMENTARY FIGURES AND TABLE

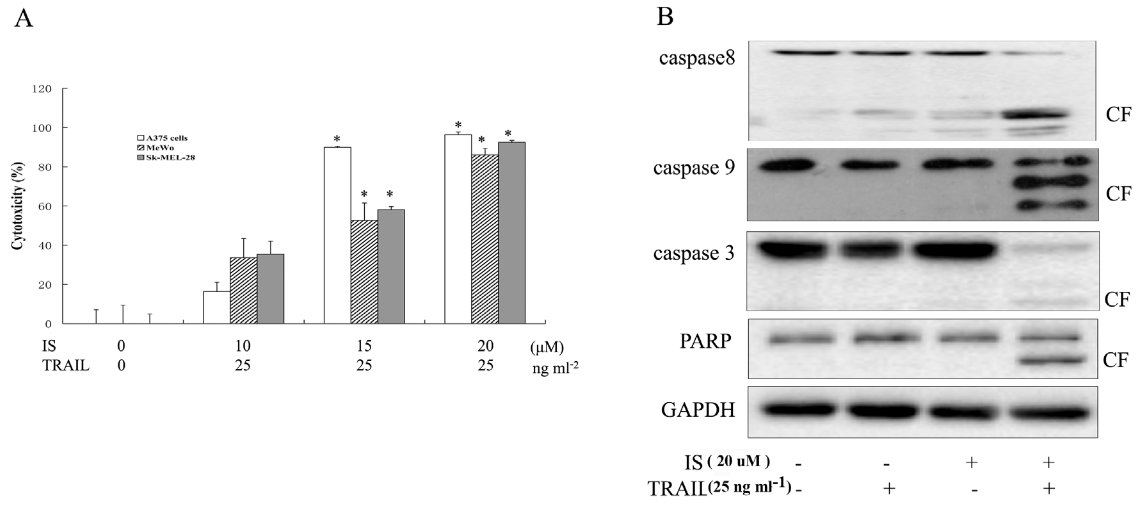

Supplementary Figure S1: Effects of IS with different concentration on TRAIL-induced apoptosis in A375, MeWo and SK-MEL-28 melanoma cells. A. Cell viability was assessed by the MTT assay. $^{*}P < 0.05$ versus control. **B.** Replicate blots of Figure 1D.

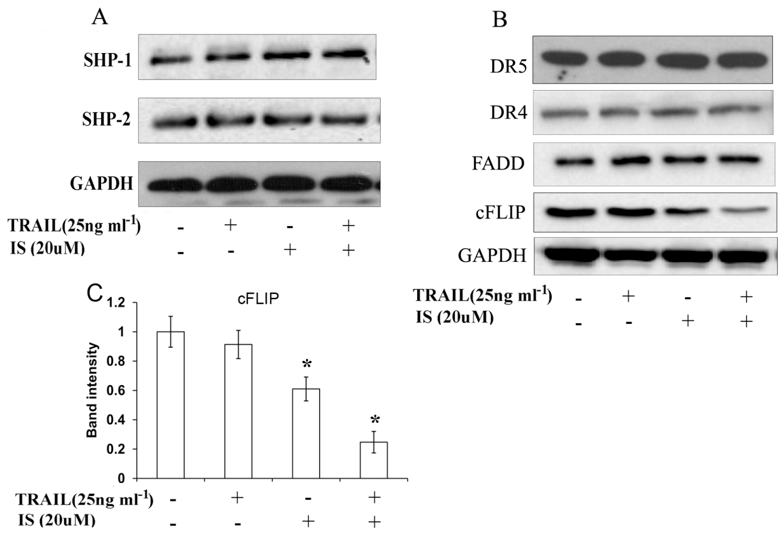

Supplementary Figure S2: Validation of cFLIP. A. The effect of IS on pSTAT3-related protein phosphatases. Whole-cell extracts were analyzed for SHP-1 and SHP-2 by western blotting. **B.** Replicate blots of Figure 2A. **C.** Band intensities were quantified using UN-SCAN-IT gel analysis software (version 6; Silk Scientific, Inc., Orem, UT, USA). The optical density for target protein was shown as a proportion of GAPDH optical density. *P<0.05 versus control.

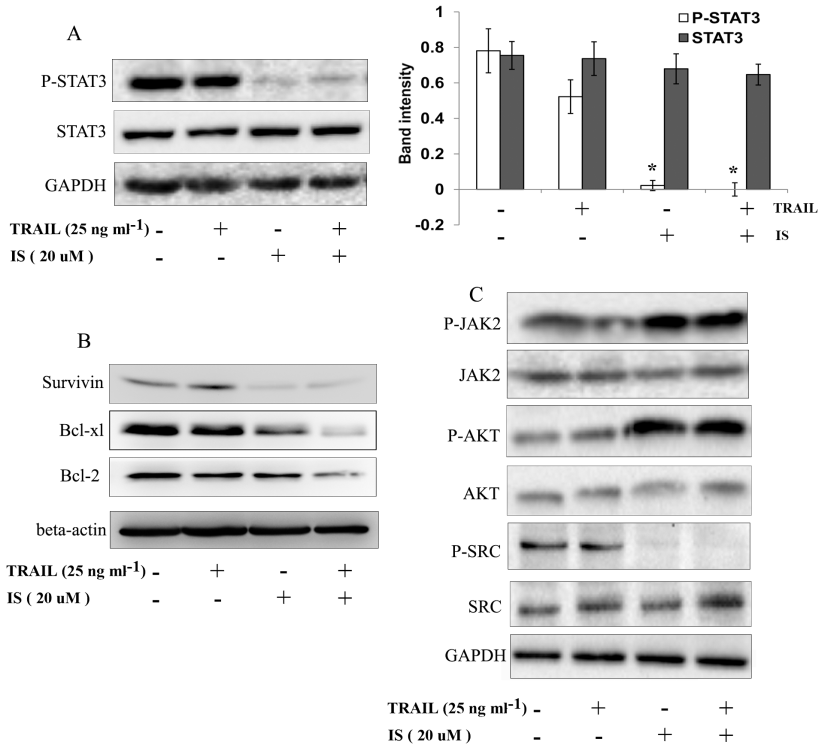

Supplementary Figure S3: Validation of STAT3 signaling. A. Replicate blots of Figure 3A.(left) Band intensities were quantified using UN-SCAN-IT gel analysis software (version 6; Silk Scientific, Inc., Orem, UT, USA). The optical density for target protein was shown as a proportion of GAPDH optical density (right). **P*<0.05 versus control. **B.** The inhibitio of IS on STAT3-activated anti-apoptotic proteins. Western blotting of A375 lysates from cells treated with IS and/or TRAIL. **C.** Replicate blots of Figure 3E.

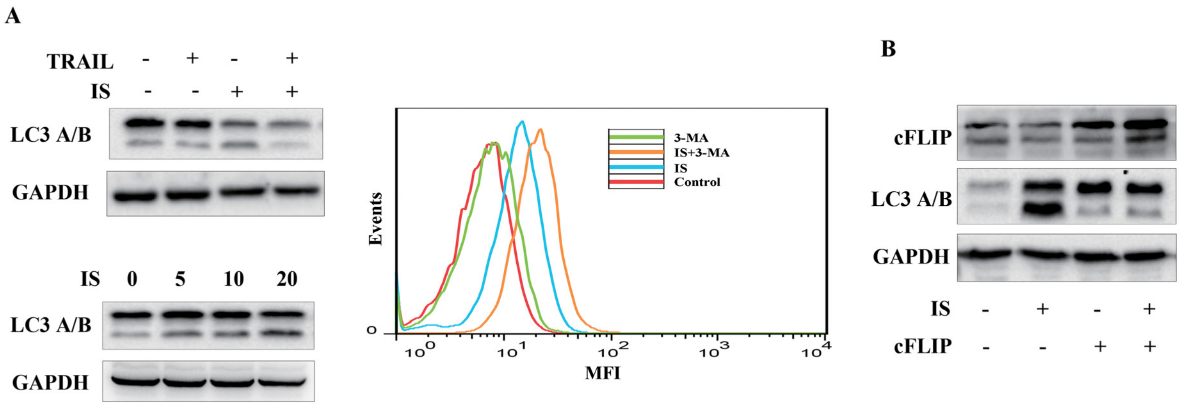

Supplementary Figure S4: FLIP-mediated autophagy regulation in IS-induced ROS accumulation. A. (Left) Whole-cell extracts were analyzed for activation of LC3 by western blotting. (Right) Fluorescence was measured using Flow cytometry for ROS. **B.** Cells were transfected with cFLIP plasmid, and cell extracts were prepared for western blot analysis of LC3. At least two independent experiments revealed largely comparable results.

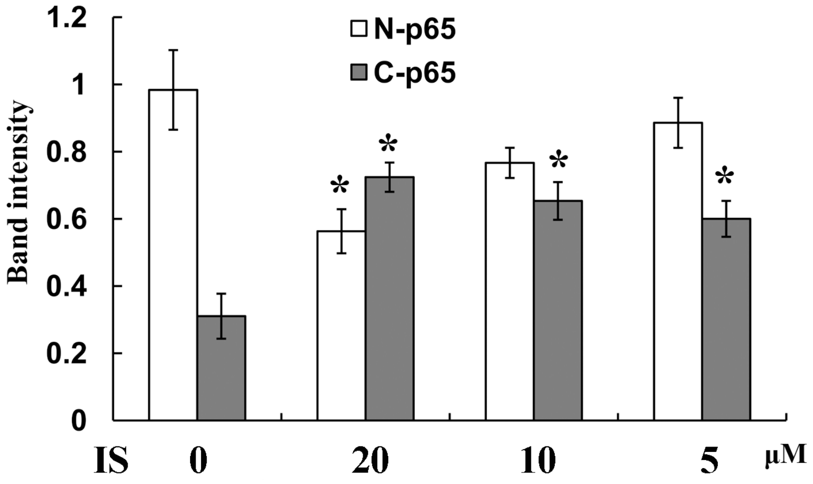

Supplementary Figure S5: Validation of p65. Band intensities of Figure 5A were quantified using UN-SCAN-IT gel analysis software (version 6; Silk Scientific, Inc., Orem, UT, USA). The optical density for target protein was shown as a proportion of GAPDH optical density (right). *P<0.05 versus control.

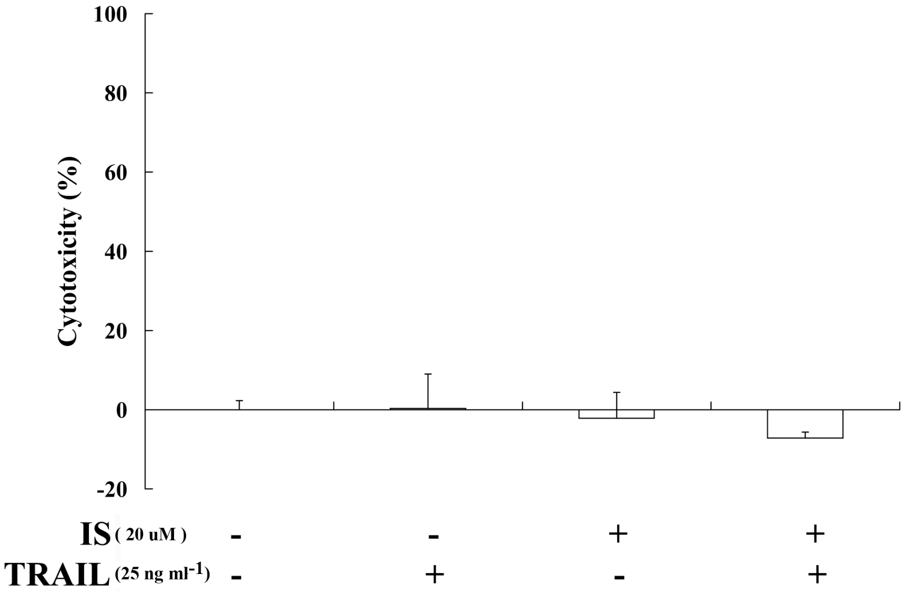

Supplementary Figure S6: The effects of IS and/or TRAIL on primary human skin fibroblast. Cell viability was assessed by the MTT assay.

Supplementary Table S1: Combination index of IS and TRAIL

TRAIL (ng/ml)	IS (µM)	Cytotoxicity	CI
25	20	0.89	0.12814
25	15	0.79	0.15670
50	20	0.93	0.09445
50	15	0.9	0.09051
